# Therapeutic options for patients with rare rheumatic diseases: a systematic review and meta-analysis

**DOI:** 10.1186/s13023-020-01576-5

**Published:** 2020-10-31

**Authors:** Tim T. A. Bender, Judith Leyens, Julia Sellin, Dmitrij Kravchenko, Rupert Conrad, Martin Mücke, Matthias F. Seidel

**Affiliations:** 1 Center for Rare Diseases Bonn, University Hospital of Bonn, Bonn, Germany; 2 Children’s University Hospital of Bonn, Bonn, Germany; 3 Radiological Department, University Hospital of Bonn, Bonn, Germany; 4 Department of Psychosomatic Medicine and Psychotherapy, University Hospital Bonn, Bonn, Germany; 5 Department of Rheumatology, Hospital Centre Biel-Bienne, Biel, Switzerland

**Keywords:** Rare disease, Pharmacotherapy, Meta-analysis, Vasculitis, Connective tissue disease, Rheumatology, Systematic review, Hunter disease (orpha: 580), Behçet’s disease (orpha: 117), ANCA-associated vasculitis (orpha: 156152), Giant cell arteritis (orpha: 397), Systemic sclerosis (orpha: 90291)

## Abstract

**Background:**

Rare diseases (RDs) in rheumatology as a group have a high prevalence, but randomized controlled trials are hampered by their heterogeneity and low individual prevalence. To survey the current evidence of pharmacotherapies for rare rheumatic diseases, we conducted a systematic review and meta-analysis. Randomized controlled trials (RCTs) of RDs in rheumatology for different pharmaco-interventions were included into this meta-analysis if there were two or more trials investigating the same RD and using the same assessment tools or outcome parameters. The Cochrane Central Register of Controlled Trials (CENTRAL), MEDLINE, Embase, and PUBMED were searched up to April 2nd 2020. The overall objective of this study was to identify RCTs of RDs in rheumatology, evaluate the overall quality of these studies, outline the evidence of pharmacotherapy, and summarize recommended therapeutic regimens.

**Results:**

We screened 187 publications, and 50 RCTs met our inclusion criteria. In total, we analyzed data of 13 different RDs. We identified several sources of potential bias, such as a lack of description of blinding methods and allocation concealment, as well as small size of the study population. Meta-analysis was possible for 26 studies covering six RDs: Hunter disease, Behçet’s disease, giant cell arteritis, ANCA-associated vasculitis, reactive arthritis, and systemic sclerosis. The pharmacotherapies tested in these studies consisted of immunosuppressants, such as corticosteroids, methotrexate and azathioprine, or biologicals. We found solid evidence for idursulfase as a treatment for Hunter syndrome. In Behçet’s disease, apremilast and IF-α showed promising results with regard to total and partial remission, and Tocilizumab with regard to relapse-free remission in giant cell arteritis. Rituximab, cyclophosphamide, and azathioprine were equally effective in ANCA-associated vasculitis, while mepolizumab improved the efficacy of glucocorticoids. The combination of rifampicin and azithromycin showed promising results in reactive arthritis, while there was no convincing evidence for the efficacy of pharmacotherapy in systemic sclerosis.

**Conclusion:**

For some diseases such as systemic sclerosis, ANCA-associated vasculitis, or Behcet's disease, higher quality trials were available. These RCTs showed satisfactory efficacies for immunosuppressants or biological drugs, except for systemic sclerosis. More high quality RCTs are urgently warranted for a wide spectrum of RDs in rheumatology.

## Background

Rare diseases (RDs) in rheumatology present a heterogeneous group of diverse syndromes, and differ in their etiology, clinical symptoms, prognosis, and outcome in clinical trials. Recently, we identified a set of various RDs in rheumatology [1], and found that they are highly prevalent when considered as a group. Most of these diseases and syndromes have a prevalence of less than one individual in 100,000, and all of them fall under the European Commission’s definition that specifies RDs as “Any disease affecting fewer than five people in 10,000” [2]. However, the sum of all identified rheumatological RDs results in a combined point prevalence of 49 in 10,000 (1). Of note, this is more than double the prevalence of ankylosing spondylitis (AS) with 18/10,000 [3], which is one of the more common diseases in the field of rheumatology. The syndromes we have previously identified as RDs in rheumatology [1] include genetic disorders, e.g., cryopyrin associated periodic syndromes (CAPS) [4], reactive arthritis [5], and diseases with unknown etiologies such as systemic sclerosis [6]. Randomized controlled trials (RCTs) and thus evidence-based pharmacotherapies are unavailable for a magnitude of these conditions.

The overall objectives of this study were to identify RCTs of RDs in rheumatology, evaluate study quality on the basis of risk of bias, elucidate the findings from pharmacotherapeutic RCTs, and summarize evidence-based recommendations.

## Methods

### Criteria for considering studies for this review

Pharmacotherapies of diseases previously identified as RDs in rheumatology with substances such as corticosteroids, antibiotics, disease modifying antirheumatic drugs (DMARD), biologicals or other immunosuppressants were included, e.g. Methotrexate, Sulfasalazine, Cyclophosphamide, Rituximab, Adalimumab, Anakinra. The classification as RDs followed the definition of the European Union, which considers a disease to be rare when < 5 out of 10,000 people are affected.

Trials were excluded if they reported findings from animals, or neonates defined by the world health organization (WHO) as children less than four weeks (28 days) old. Studies with less than 10 participants per study arm in the final analysis were excluded. We considered only rheumatic and musculoskeletal diseases as defined by EULAR and the Orphanet classification of rare rheumatic diseases as ORPHA: 182231 (compare Leyens et al. [1] and Orphanet).

### Search methods for identification of studies

**Fig. 1:**
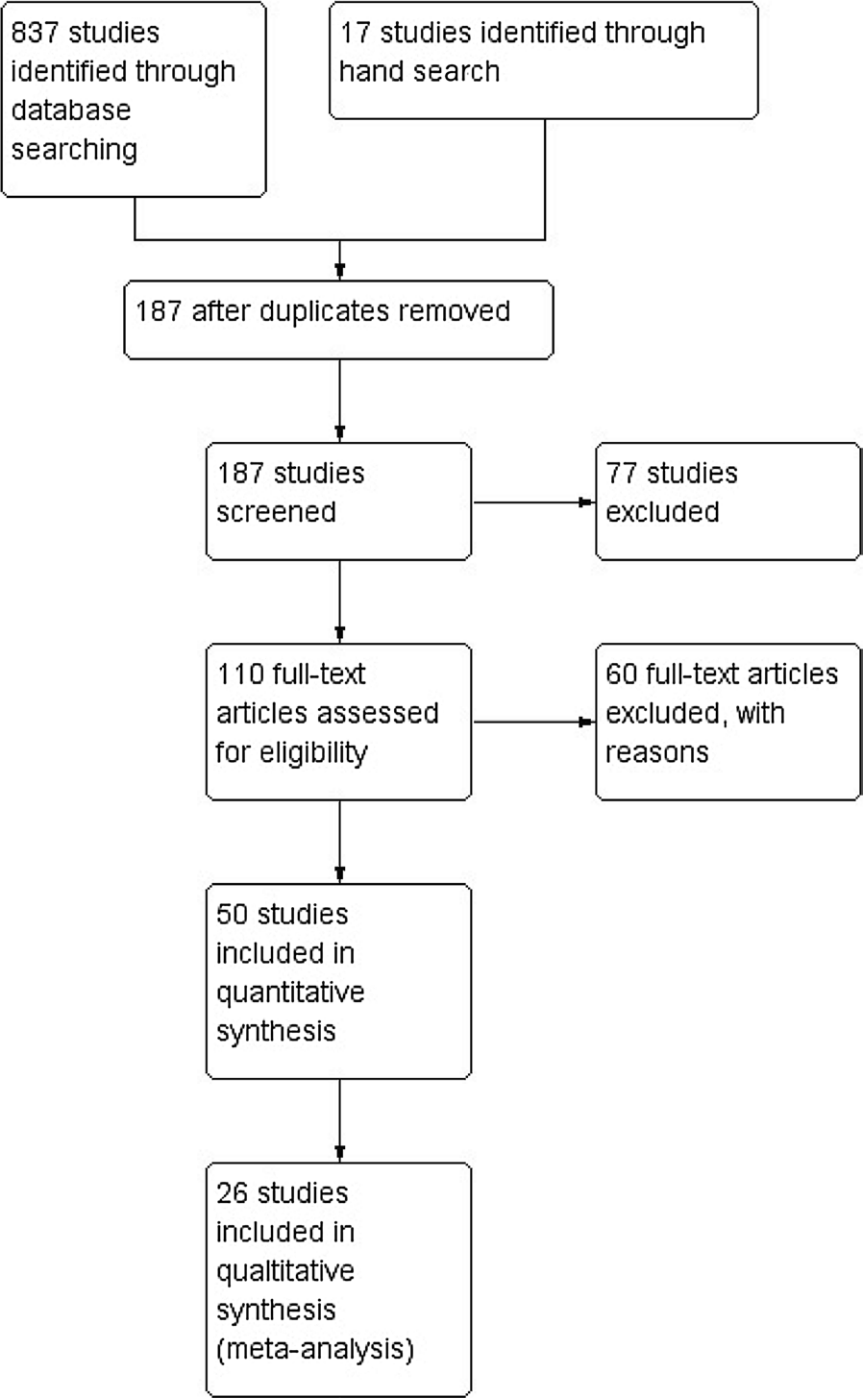
Study flow diagram of clinical trials included in this meta-analysis. Predefined criteria were used to select for high quality interventional trials. 26 studies were finally included

To identify RCTs, we developed a comprehensive search strategy for each electronic database. We only searched English-language literature. For quality control, we selected relatively new, but well-known publications of high relevance (e.g. “Trial of Tocilizumab in Giant-Cell Arteritis”, Stone et al. [7] and “Apremilast for Behçet’s syndrome—a phase 2, placebo-controlled study”, Hatemi et al. [8]) and confirmed that the publication was covered by our search strategy. This method was used to validate the accuracy of our literature search. To gather all relevant evidence, we applied a broad search strategy (see the Additional file 1: S1 for a precise description). We used this approach because we discovered that a more precise search strategy, as recommended in the Cochrane handbook, did not yield all results we expected, and we concluded that it might not be applicable for rare disease (section Discussion). In the next step, irrelevant studies were excluded after screening the abstract according to inclusion and exclusion criteria (Fig. 1). The remaining studies were read in full, but most of these studies considered for full text review could not be included in our meta-analysis because the outcome measures were not standardized and comparable to one another (Fig. 1).

#### Electronic searches

We searched the following electronic databases:

- PubMed up until 2nd April 2020.
- Cochrane Central Register of Controlled Trials (CENTRAL) up until 2nd April 2020.
- MEDLINE (OVID) up until 2nd April 2020.
- Embase up until 2nd April 2020.

The applied search strategies for each database can be found in the Additional file 1: S1. In addition, we searched PubMed by hand up until 7th August 2020.

#### Searching other resources

Other resources were not considered.

### Data collection and analysis

#### Selection of studies

We retrieved, in full, studies with abstracts referring to treatment for RDs in rheumatology.

#### Data extraction and management

We extracted data from each included RCT into an individually designed spreadsheet (the data extraction form) containing the categories: study design, study setting, exclusion and inclusion criteria, study size, patient demographics, and outcome measures.

One author (TB) extracted the data using the standardized data extraction form and reviewed the data from the studies (Fig. 1).

#### Assessment of risk of bias in included studies

**Fig. 2:**
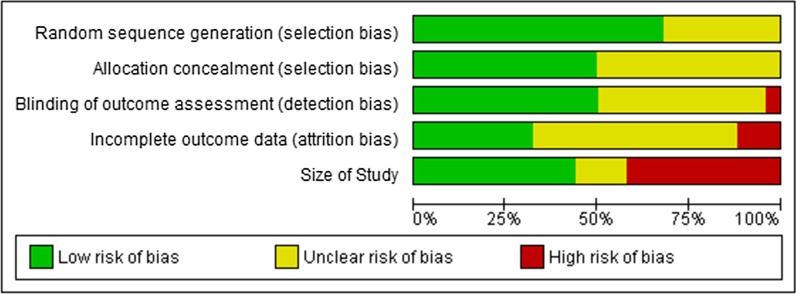
Risk of bias graph

**Fig. 3:**
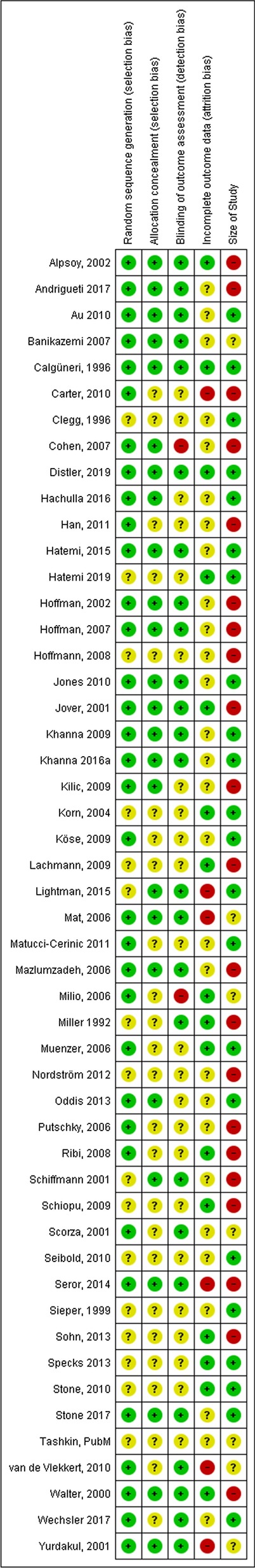
Risk of bias summary: review authors' judgements about each risk of bias item for each of the 50 included studies

One author (TB) assessed risk of bias via the Cochrane risk of bias tool (Figs. 2 and 3) for each study, using the criteria outlined in the Cochrane Handbook for Systematic Reviews of Interventions [9], and presented the results to the other authors, who gave their opinion with regard to the first author’s assessment. In case of disagreements, they were resolved by discussion and consensus (JL, DK, RC, MM, MFS). The results are presented in the risk of bias graph (Fig. 2) which reviews the authors’ judgements about each risk of bias item, shown as percentages across all included studies, and the risk of bias summary (see Fig. 3).We assessed the following for each study:

#### Random sequence generation

We classified the method used to generate the allocation sequence as follows: “low risk of bias” for any truly random process, e.g. random number table or computer random number generator, and “unclear risk of bias” when the method used to generate sequence was not available in the full text.

#### Allocation concealment

The method used to conceal allocation to interventions prior to assignment determines whether intervention allocation could have been determined in advance of, or during recruitment, or changed after assignment. We assessed the methods as follows: “low risk of bias” (e.g. telephone or central randomization, or consecutively numbered or sealed envelopes) and “unclear risk of bias” when the method was not clearly stated.

#### Blinding of outcome assessment

We assessed the methods used to blind participants and outcome assessors from the knowledge of which intervention a participant received. We assessed the methods as follows: “low risk of bias” when the study states that it was blinded, and describes the method used to achieve this blinding, e.g., the study used medication of identical appearance, and “unclear risk of bias” when the study states that it was blinded but does not provide a description of how this was achieved.

#### Incomplete outcome data

We assessed the methods to handle incomplete outcome data as follows: “low risk of bias” (less than 10% of participants did not complete the study).

#### Size of study

Studies dealing with RDs usually have smaller sample sizes due to the inherent small number of available participants. For evaluation of study size, we took that into account and assessed studies as being at “low risk of bias” with 50 participants or more per treatment arm; “unclear risk of bias” with 25–49 participants per treatment arm; and “high risk of bias” with fewer than 25 participants per treatment arm.

### Statistical methods

To generate the forest plot graphs and calculate the combined odds ratio (OR) or differences in mean in this meta-analysis, we used the Cochrane recommended program Review Manager (RevMan) 5. The statistical algorithms used by the program are described elsewhere [10]. In brief, we selected in RevMan5 to calculate odds ratios for dichotomous outcomes and differences in mean for continuous outcomes for individual study estimates, which are depicted in the forest plots as box plots spanning the 95% confidence interval (CI), while the summarized odds ratios/ differences of mean from several studies are depicted as rhombi spanning the 95% CI. For estimation of heterogeneity (fixed effect model), the program calculates Chi^2^ (with *p* value and degrees of freedom df) and I^2^ values. Due to the low number of studies per intervention and/or outcome measure, the power of Chi^2^ is however limited. Chi^2^ with *p* value and I^2^ are given in the figures for all studies testing the same treatment, as well as for a group of treatments testing the same outcome effect. Overall effect is estimated by the RevMan5 program with a Z-test, whose result with its *p* value is given in the figures for each intervention, as well as for groups of interventions for the same outcome measure. To standardize the Results section, we described individual results by comparing either odds ratio or mean difference, depending on outcome measures (dichotomous vs. continuous, respectively). Due to the limited number of available studies, heterogeneity and overall effect measures have to be considered with caution.

## Results

In total, we screened 187 trials that examined drug interventions for RDs in rheumatology. 50 RCTs met the inclusion criteria for this systematic review (Fig. 1). Study characteristics are shown in the Additional file 1: S1.

The number of participants varied across studies (22 to 576 participants). The previously defined literature key words indeed identified high-quality trials and excluded small case reports. The paucity of data from high quality studies on RD in rheumatology became evident during this evaluation process. Because of the small number of studies and their inhomogeneity, meta-analysis was possible for only 26 studies dealing with six diseases: Hunter syndrome, Behçet’s syndrome, giant cell arteritis, ANCA-associated vasculitis, reactive arthritis and systemic sclerosis.

### Hunter syndrome

Hunter syndrome (mucopolysaccharidosis type II) is a genetic disorder caused by a deficiency of iduronate 2-sulfatase. This defect results in excessive storage of heparan and dermatan in lysosomes [11]. In total we included two trials in our quantitative synthesis.

**Fig. 4:**
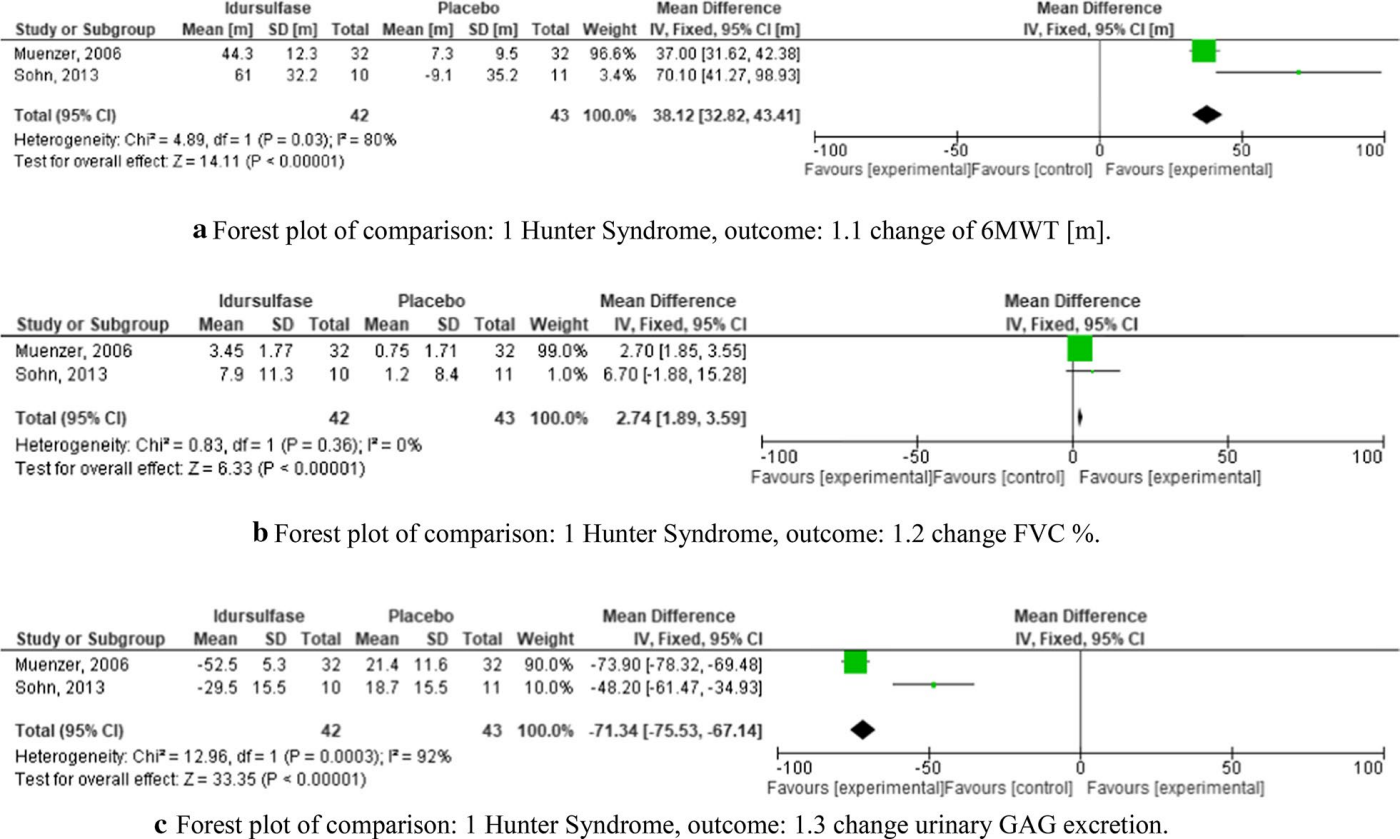
A. Forest plot of comparison: 1 Hunter Syndrome, outcome: 1.1 change of 6MWT [m]. B. Forest plot of comparison: 1 Hunter Syndrome, outcome: 1.2 change FVC %. C: Forest plot of comparison: 1 Hunter Syndrome, outcome: 1.3 change urinary GAG excretion

We analyzed data from two RCTs with a total of 85 participants [12, 13] (Fig. 4). Risk of bias for these studies is presented in Fig. 3. Both studies compared idursulfase as an enzyme replacement to a placebo and yielded similar results. The primary outcome parameters were the change in the six-minute-walking-test (6MWT) (Fig. 4a), the percent change of forced vital capacity (Fig. 4b), and the change in urinary glycosaminoglycan (GAG) excretion (Fig. 4c). When looking at the change in the 6MWT, the authors detected a combined mean difference of 38.12 (CI 32.82–43.41) in favor of the enzyme replacement therapy (Fig. 4a). The other two outcome parameters also showed a significant benefit of idursulfase. (Fig. 4b,c).

Taken together, there is solid evidence for idursulfase as a treatment for Hunter syndrome with an overall good safety profile. Most adverse events were mild, such as mild respiratory infections, headache, or urticaria and skin rash, which could be controlled easily.

### Behçet’s syndrome

Behçet's syndrome is a systemic vasculitis, which may affect almost every vascularized area of the body. There is a close correlation between the geographical distribution of HLA-B51 and its prevalence [14], but the etiology is unknown. In total we included ten trials in our quantitative synthesis.

**Fig.5:**
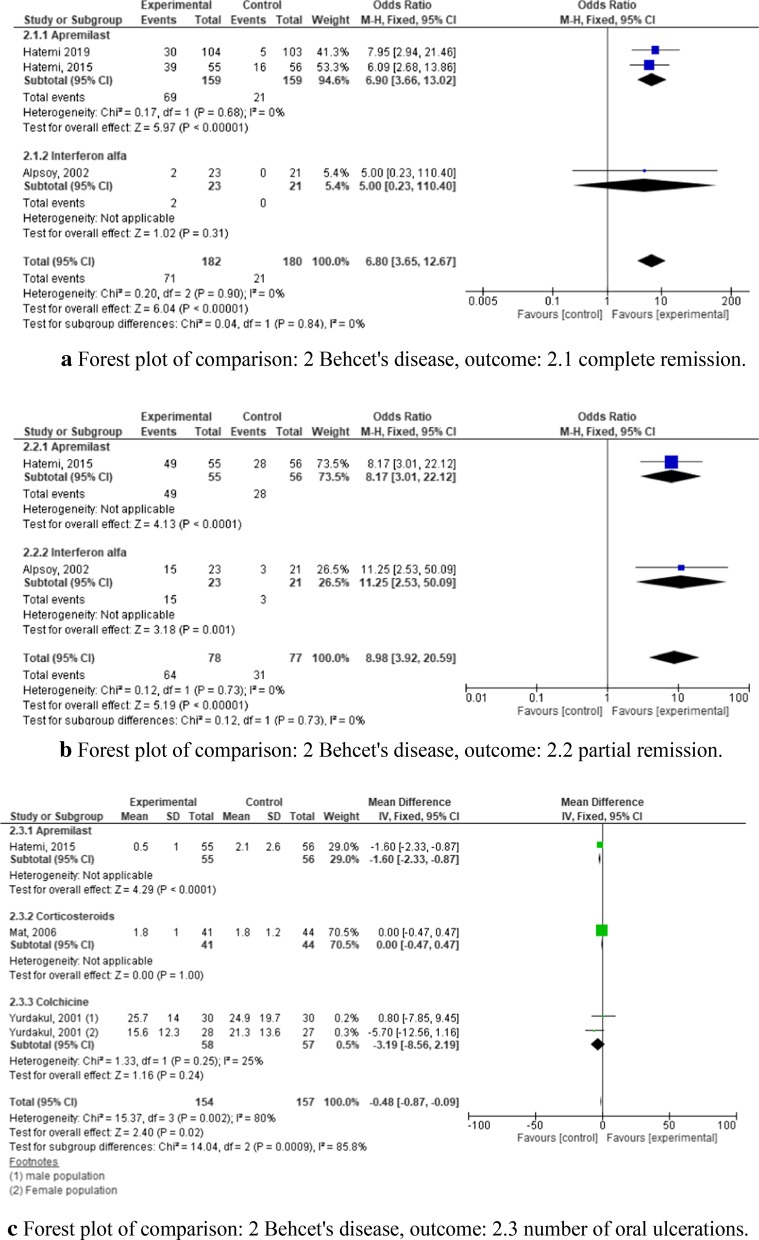
**a** Forest plot of comparison: 2 Behcet's disease, outcome: 2.1 complete remission. **b** Forest plot of comparison: 2 Behcet's disease, outcome: 2.2 partial remission. **c** Forest plot of comparison: 2 Behcet's disease, outcome: 2.3 number of oral ulcerations

We analyzed data from five RCTs for Behçet’s syndrome, which tested four interventions: the novel Phosphodiesterase-4 (PDE4) inhibitor apremilast (two studies), corticosteroids, interferon-α (IF-α), and colchicine [8, 15–18]. Risk of bias for these studies is presented in Fig. 3. We compared three outcome parameters, which were the number of total remissions (sustained absence of any lesions during treatment) (Fig. 5a), partial remissions (when total remission was not achieved) (Fig. 5b), and the number of oral ulcerations (Fig. 5c). Only apremilast and IF-α were analyzed for total or partial remission (Fig. 5a,B). With regard to total remission, both compounds were effective. The combined odds ratio of the two trials investigating apremilast with regard to complete remission was 6.90 (CI 3.66–13.02) and slightly higher and with a narrower CI compared to the odds ratio of IF-α with 5.00 (CI 0.23–110.4). The odds ratios for the outcome of partial remission were even higher in both studies (Fig. 5B). With regard to oral ulcerations, three of the five studies were analyzed, which tested apremilast, corticosteroids, and colchicine. None of those regimens were significantly superior to the placebo (mean difference −0.48, CI −0.87 to −0.09) with regard to oral ulcerations (Fig. 5c).

In summary, apremilast and IF-α showed promising results with regard to total and partial remission, but not with regard to oral ulcerations. The most frequent adverse events of apremilast were diarrhea, nausea, and headache. Common adverse events of IF-α as reported by Alpsoy et al. were mild flu like symptoms [15]. Concerning corticosteroids and colchicine the authors reported similar adverse events in the treatment and controlled groups and attested an overall good safety profile [16, 17].

### Giant cell arteritis

Giant cell arteritis (GCA) is a vasculitis of large-sized and medium-sized vessels, frequently causing critical ischemia. The disease may also affect the ocular nerve, often leading to irreversible loss of vision [19]. In total we included six trials in our quantitative synthesis and could include all of them in our meta-analysis.

**Fig. 6:**
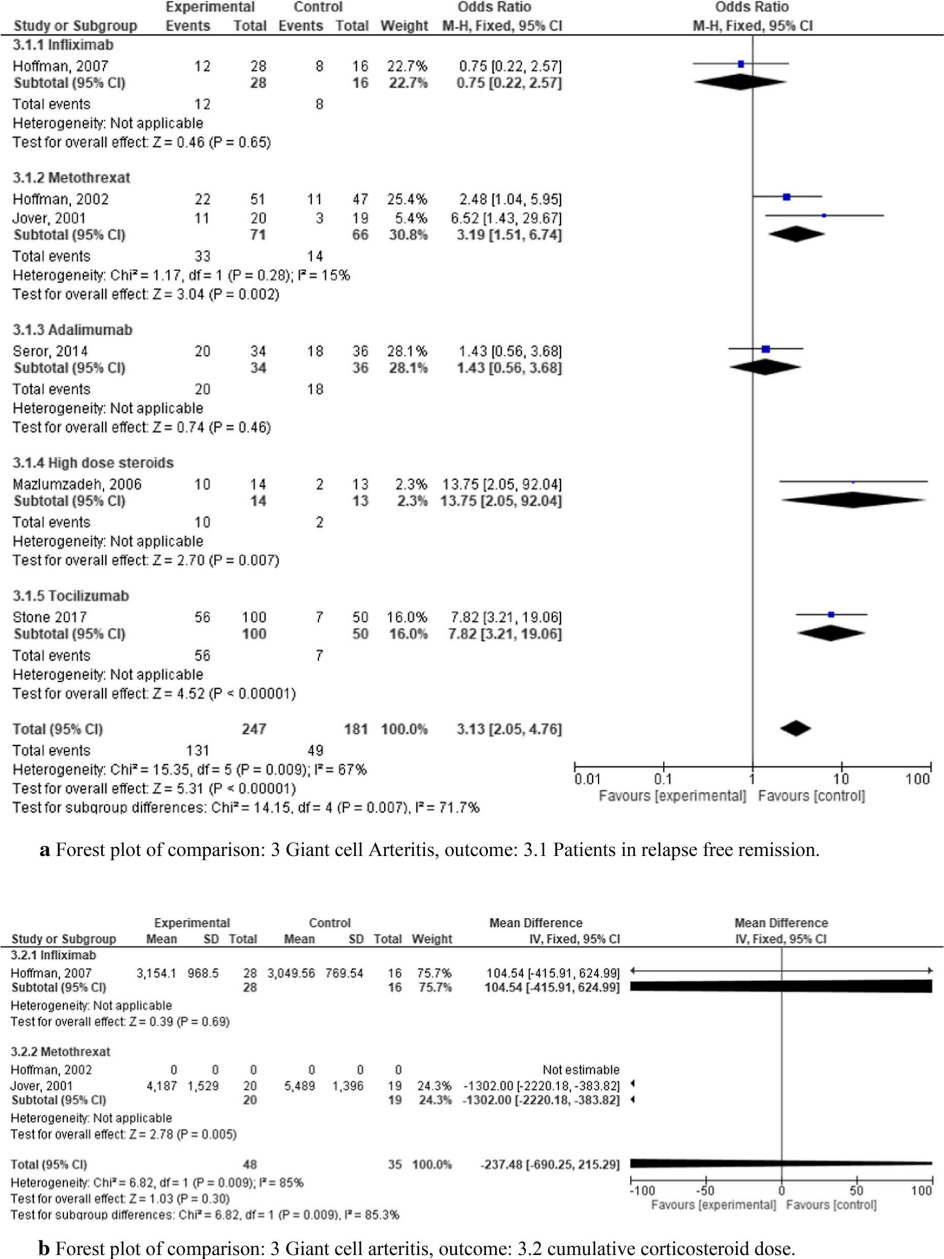
**a** Forest plot of comparison: 3 Giant cell Arteritis, outcome: 3.1 Patients in relapse free remission. **b** Forest plot of comparison: 3 Giant cell arteritis, outcome: 3.2 cumulative corticosteroid dose

For our study we identified six RCTs for GCA. Risk of bias for these studies is presented in Fig. 3. Outcome parameters were relapse-free remission (Fig. 6a) and the steroid-sparing effect as determined by cumulative corticosteroid dose (Fig. 6b). These RCTs examined five different regimens including infliximab, methotrexate (MTX, two studies), adalimumab, tocilizumab, and high dose corticosteroids [7, 20–24]. All six studies analyzed the outcome parameter relapse-free remission (Fig. 6A). Two of them (Jover et al. [23] and Hoffman et al. [20]) compared MTX combined with glucocorticoids against glucocorticoids and placebo, and showed no difference for relapse-free remission after 12 months. Figure 6a shows that the results of Jover et al. [23] were more promising in terms of the odds ratio (OR 6.52, CI 1.43–28.67), but due to the smaller sample size had less of an impact in the overall odds ratio of both studies of only 3.19 (CI 1.51–6.74). The most recent RCT (Stone et al. [7]) included the largest number of patients (n = 150) and examined tocilizumab versus glucocorticoid alone in GCA. 56 out of 100 patients treated with tocilizumab reached the primary outcome parameter of relapse-free remission as compared to only 7 patients out of 50 in the glucocorticoid group with an odds ratio of 7.82 (CI 3.21–19.06) (Fig. 6a). One study tested high dose steroids with regard to effect on relapse-free remission [24] with a positive outcome (OR 13.75, CI 2.05–92.04). In contrast, the remaining two studies, which tested treatment with infliximab and adalimumab, did not yield statistically significant results with regard to relapse-free remission [20, 22]. In summary, our analysis showed a combined odds ratio of 3.13 (CI 2.05–4.76) of treatments with regard to relapse free remission of GCA.

Three of the six studies analyzed the regimen also with regard to their steroid-sparing effects (Fig. 6b). The two studies investigating MTX (Hoffmann et al. 2002, Jover et al. [20, 23] showed no benefit for this outcome. The third study analyzed the effect of infliximab on the effect of steroid dose (Hoffmann et al. [20]). It demonstrated that patients with GCA treated with infliximab received numerically higher steroid doses than patients treated with glucocorticoid alone (mean difference of 104.54, corresponding to mean doses of 3154.1 mg vs. 3049.56 mg), but with a very wide CI of −415.91–624.99 (Fig. 6b). Accordingly, the *p* value for the overall effect of infliximab is far beyond the significance threshold, with *p* = 0.69.

Overall, only tocilizumab shows some promise as a treatment for GCA, with the measured outcome parameter being relapse-free remission. The overall safety profile for the five tested agents was promising. Seror et al. [22] reported in their trial testing Adalimumab that “serious adverse events occurred in five (14.7%) patients on adalimumab and 17 (47.2%) on placebo” [22]. With regard to tocilizumab, the patients receiving the treatment reported less severe events than patients in the placebo group [7].

### ANCA-associated vasculitis

ANCA-associated vasculitides (AAV) are a group of vasculitides comprising microscopic polyangiitis (MPA), granulomatosis with polyangiitis (GPA), and eosinophilic granulomatosis with polyangiitis (EGPA). According to the revised Chapel Hill Consensus Conference Nomenclature of vasculitides, AAV is defined as a necrotizing vasculitis with few or no immune deposits, predominantly affecting small blood vessels, with the presence of ANCA-autoantibodies [25]. In total we included six trials in our quantitative synthesis.

**Fig. 7:**
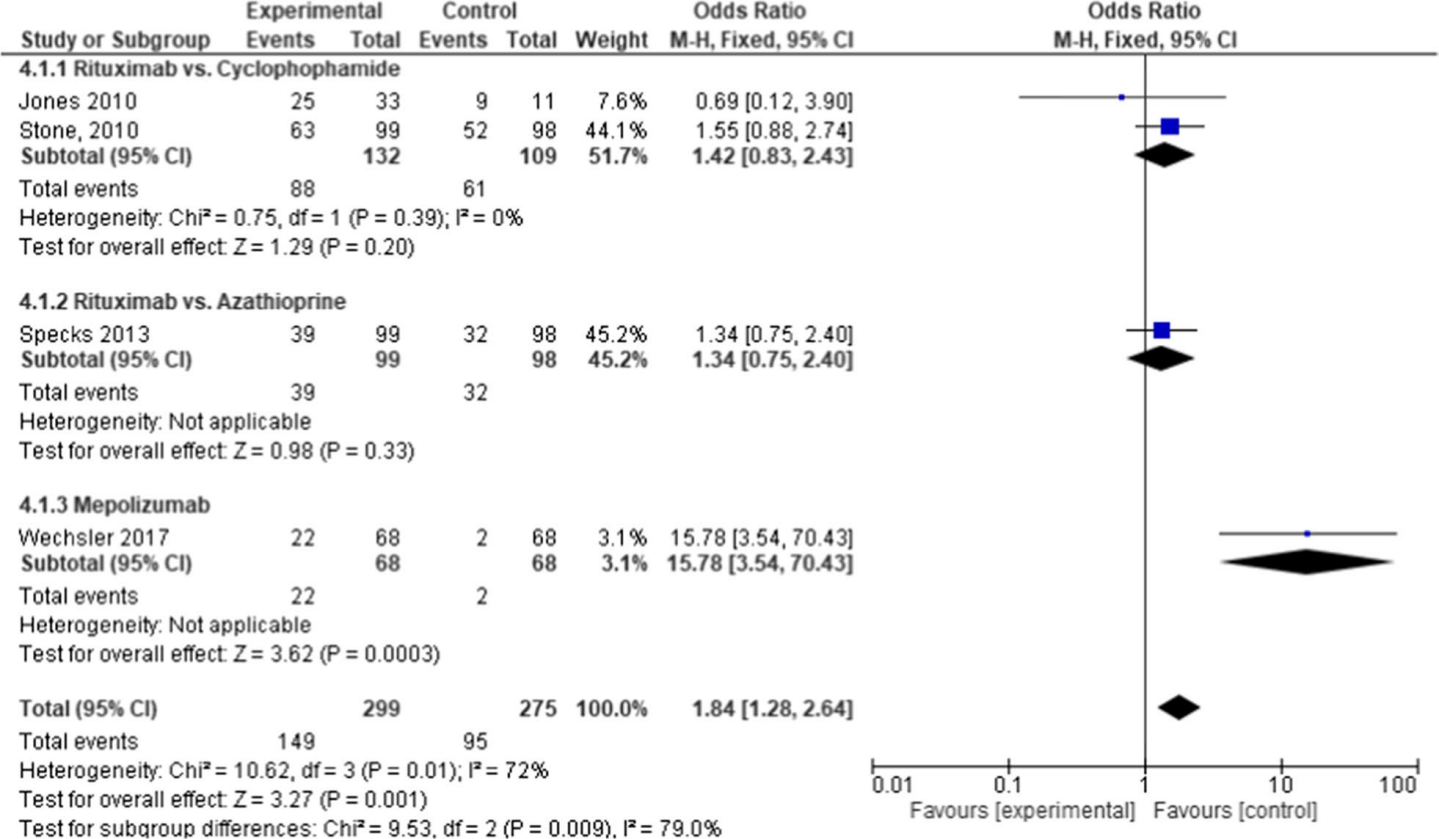
Forest plot of comparison: 4 ANCA-associated vasculitis, outcome: 4.1 total remission

We identified and included four trials for meta-analysis. Risk of bias for these studies is presented in Fig. 3. Except for one study (Wechsler et al. [26]), which focused only on EGPA, the other studies included patients with GPA and MPA. All four RCTs analyzed remission defined as reduced disease activity measured by Birmingham Vasculitis Activity Score (Fig. 7). Of those four, two non-inferiority RCTs examined rituximab (RTX) versus cyclophosphamide (CYC) [27, 28], and a third one examined RTX versus azathioprine combined with CYC [29]. In comparison to both CYC and azathioprine, RTX was similarly effective, with a combined odds ratio of RTX versus CYC of 1.42 (CI 0.83–2.43), and versus azathioprine of 1.34 (CI 0.75–2.40). RTX was slightly better tolerated in comparison to CYC. The fourth RCT examined mepolizumab (a novel anti-interleukin-5 monoclonal antibody) + stable dose of glucocorticoids, against glucocorticoids + placebo [26]. 22 out of 68 patients (32%) in the mepolizumab group achieved complete remission as compared to 2 out of 68 patients (3%) in the placebo group (odds ratio 15.78 [CI 3.54–70.43]).

Taken together, RTX was equally effective as both CYC and azathioprine, while mepolizumab in addition to glucocorticoids improved the efficacy of the latter alone.

In terms of safety, RTX seems to be as tolerable as other common agents such as the tested CYC and azathioprine. Specks et al. [29] reported that concerning total adverse events, serious adverse events, or non–disease-related adverse events the different treatment groups (RTX vs. azathioprine + CYC). Jones et al. [27] stated: “Severe adverse events occurred in 14 patients in the rituximab group (42%) and 4 patients in the control group (36%) (P = 0.77).” In another study comparing RTX to CYC, Stone et al. [28] found no significant differences between the treatment groups with respect to rates of adverse events.

### Reactive arthritis

Reactive arthritis (ReA) is a spondyloarthropathic disorder characterized by inflammation of the sacroiliacal and facet joints occurring after gastrointestinal or genitourinary infections [30]. ReA is caused by a variety of arthritogenic bacteria and is usually non-erosive. In total we included four trials in our quantitative synthesis of which one could not be included in our meta-analysis because of incomparable outcome measures.

**Fig. 8:**
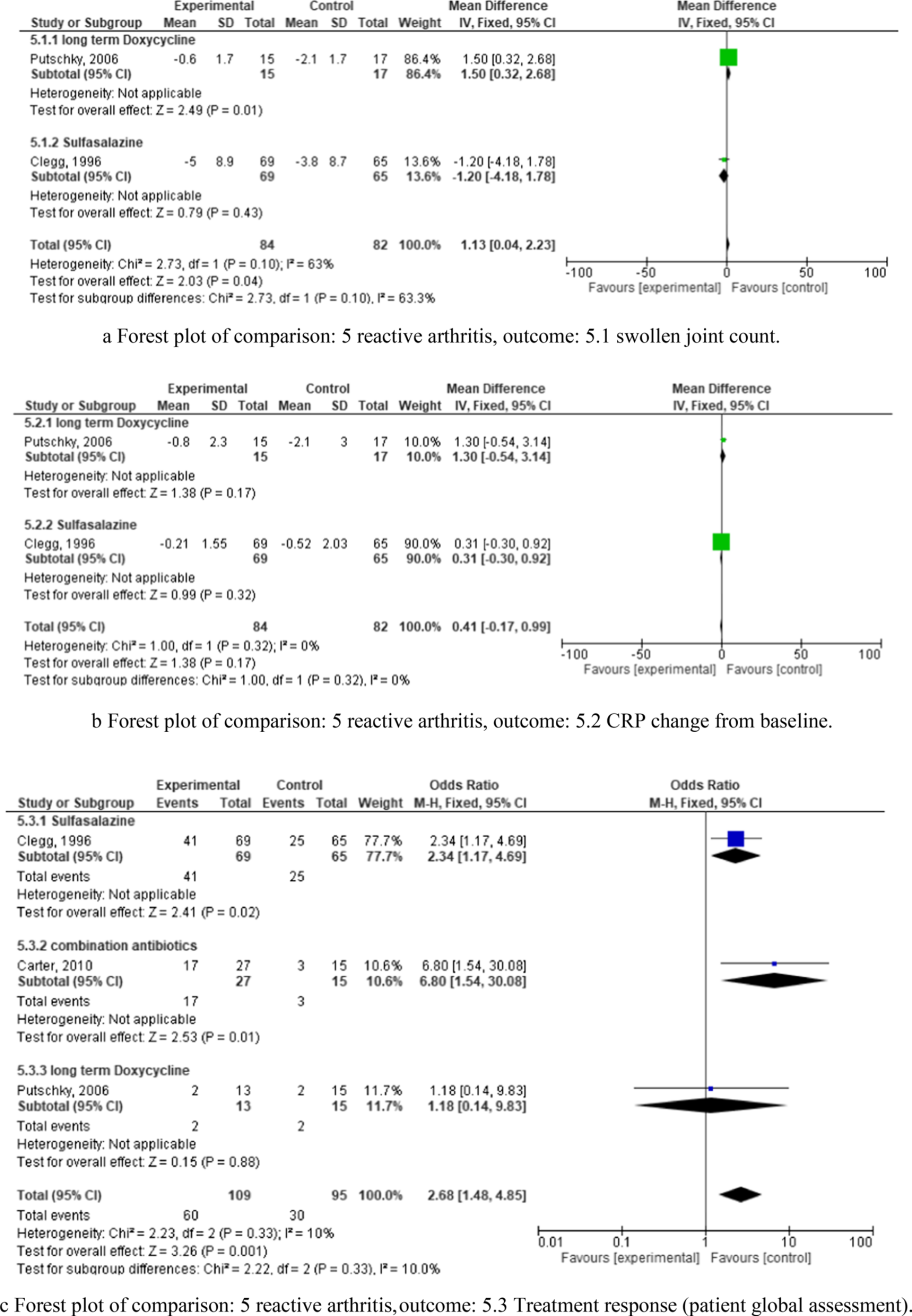
**a** Forest plot of comparison: 5 reactive arthritis, outcome: 5.1 swollen joint count. **b**. Forest plot of comparison: 5 reactive arthritis, outcome: 5.2 CRP change from baseline. **c** Forest plot of comparison: 5 reactive arthritis, outcome: 5.3 Treatment response (patient global assessment)

Three RCTs were analyzed, which tested doxycycline, sulfasalazine, and a mixture of rifampicin and azithromycin [31–33]. Risk of bias for these studies is presented in Fig. 3. Two of them (doxycycline and sulfasalazine, respectively) analyzed three outcome parameters: swollen joint count (Fig. 8a), CRP changes (Fig. 8b), and disease activity determined by patient global assessment (Fig. 8c). They did not show effects regarding CRP change and swollen joint count (Fig. 8a,b). The third RCT (Carter et al., 2010 [33]) analyzed a mixture of rifampicin and azithromycin, and only with regard to disease activity, which was also analyzed by the other two RCTs (Fig. 8c). Patient global assessment as an outcome treatment response parameter was significant only for the latter study [33] testing the rifampin/azithromycin mixture. The overall odds ratio of all three studies with regard to disease activity was 2.68 (CI 1.48–4.85).

In summary, rifampicin and azithromycin in combination showed some promise for the treatment of ReA. The safety profile of these agents were good and comparable to the usual adverse events for antibiotics. The most common adverse events were gastrointestinal symptoms [33].

### Systemic sclerosis

Systemic sclerosis (SSc) is a complex connective tissue disease of yet unknown etiology with skin and multiorgan involvement. This potentially devastating disease is associated with Raynaud’s phenomenon (RP), digital ulceration and, frequently, pulmonary involvement [34]. In total we included 13 trials in our quantitative synthesis.

**Fig. 9:**
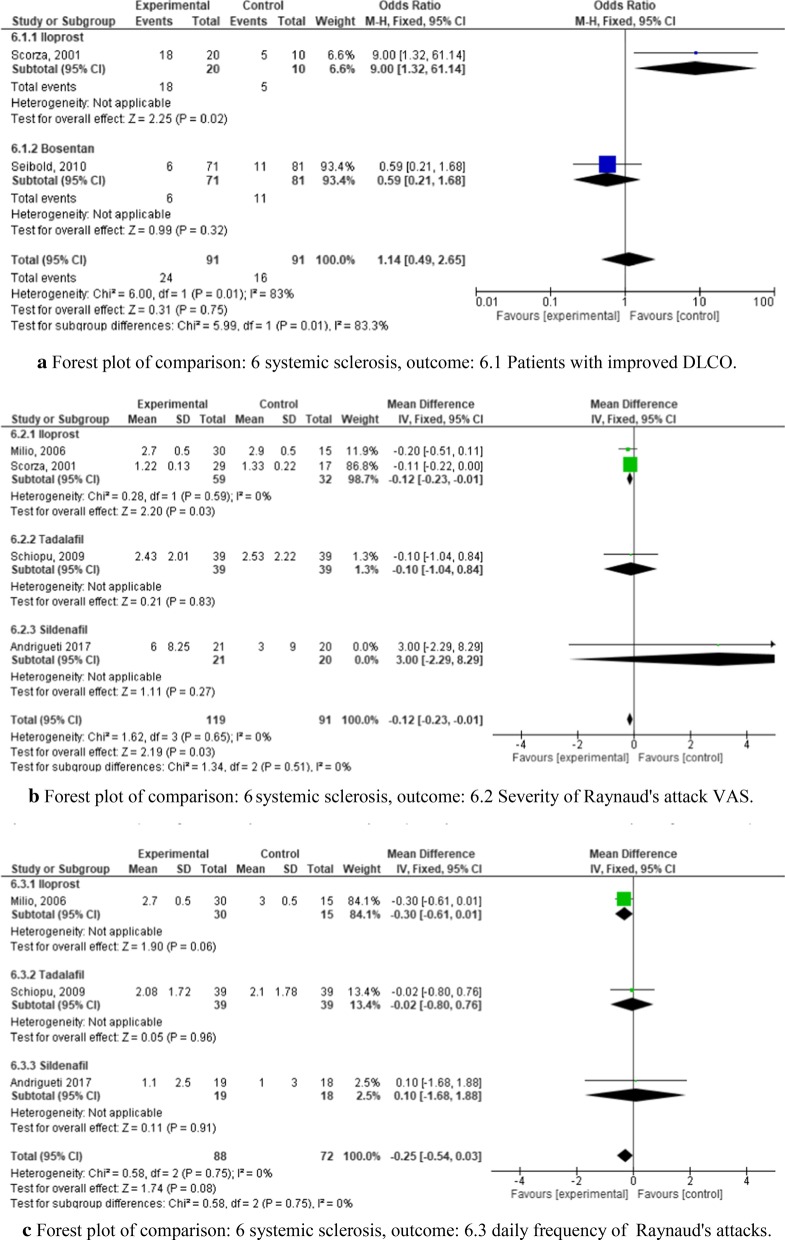

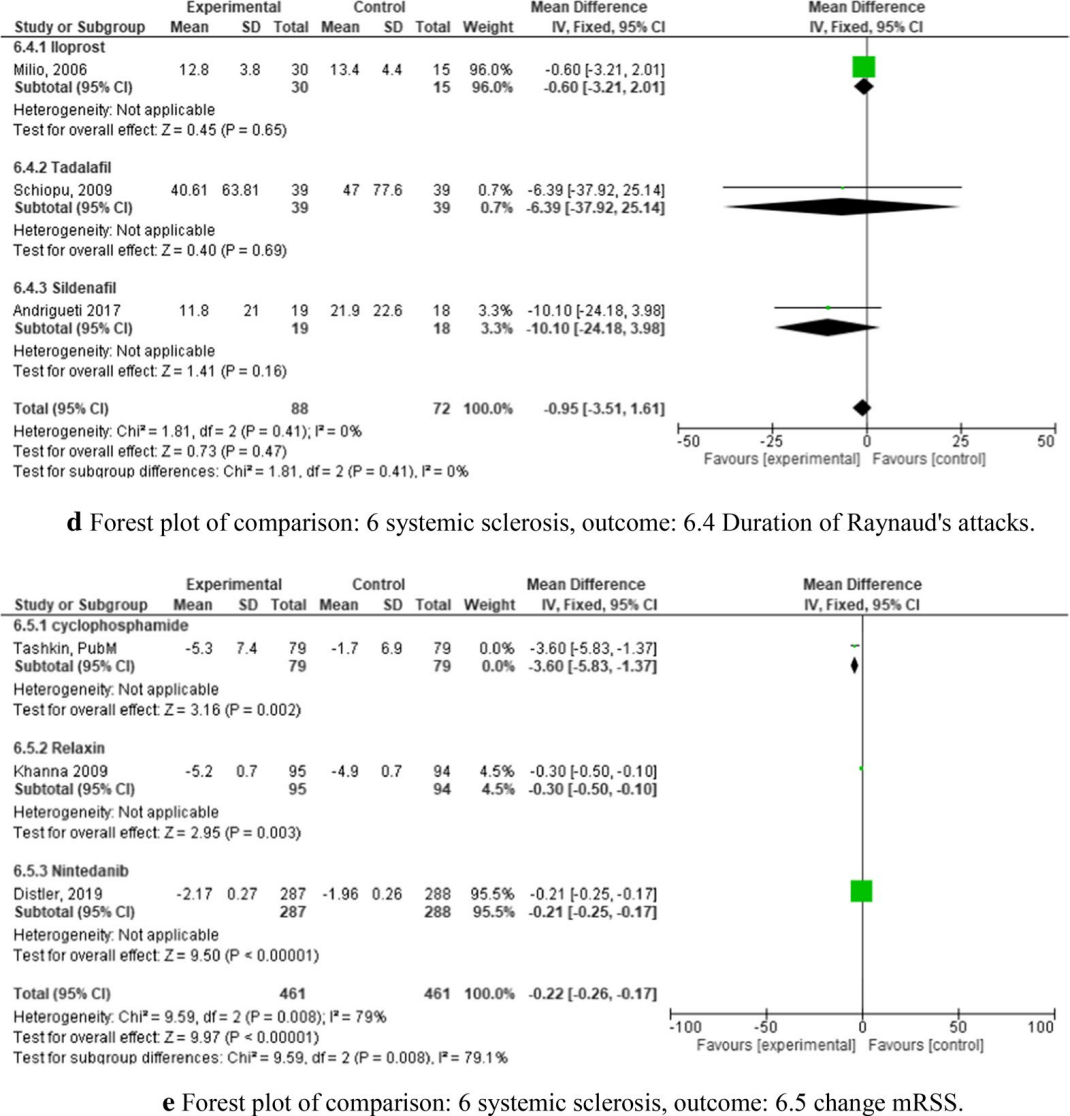
**a** Forest plot of comparison: 6 systemic sclerosis, outcome: 6.1 Patients with improved DLCO. **b** Forest plot of comparison: 6 systemic sclerosis, outcome: 6.2 Severity of Raynaud's attack VAS. **c** Forest plot of comparison: 6 systemic sclerosis, outcome: 6.3 daily frequency of Raynaud's attacks. **d** Forest plot of comparison: 6 systemic sclerosis, outcome: 6.4 Duration of Raynaud's attacks. **e** Forest plot of comparison: 6 systemic sclerosis, outcome: 6.5 change mRSS

We identified six RCTs to include in our meta-analysis which analyzed iloprost [35, 36], bosentan [37], tadalafil [38], sildenafil [39], CYC [40], relaxin [41], and nintedanib [42], each versus placebo. Risk of bias for these studies is presented in Fig. 3. Three major outcome parameters were analyzed: pulmonary diffusing capacity (DLCO, Fig. 9a), RP (Fig. 9b–d), and skin induration as determined by the modified Rodnan-Skin-Score (mRSS, Fig. 9e).

Two studies with respect to DLCO as primary outcome parameter found that iloprost appeared to be more effective than bosentan (odds ratio 9.00 (CI 1.32–61.14) compared to 0.59 (CI 0.21–1.68)). However, the bosentan trial included a higher number of patients (152 vs. 30), and this resulted in a combined odds ratio of only 1.14 (CI 0.49–2.65).

Four trials analyzed the frequency (Fig. 9b), duration (Fig. 9c), and severity of RP (Fig. 9d)—two testing iloprost, one testing tadalafil, and one sildenafil, each versus placebo. Surprisingly, the results did not show a significant improvement in any of the three outcome parameters.

Three RCTs examined the effect on skin involvement after treatment with CYC, nintedanib, or relaxin against placebo, respectively [40–42] (Fig. 9e). Patients receiving relaxin and nintedanib had only a minimal change in mRSS compared to the placebo groups (−3.6 (CI −5.83 to −1.37) and −0.21 (CI −0.25 to −0.17), respectively). The CYC trial showed a slightly higher change in mRSS, with a mean difference between the groups of −3.6 (CI −5.83 to −1.37). These values resulted in a low combined mean difference in terms of change in mRSS of the three studies of −0.22 (CI −0.26 to −0.17) (Fig. 9e).

To sum it up, there is only weak evidence for some of the tested interventions for systemic sclerosis, and only for certain outcome measures. RP was not positively affected in any of the RCTs analyzed.

For nintedanib, the most frequently reported adverse event was diarrhea which occurred in circa 75% of patients [42]. In the study evaluating relaxin it was shown that both doses tested were associated with an increase in creatinine clearance [41]. Schiopu et al. [38] reported no serious adverse events in the tadalafil and the placebo group. The most common adverse event for tadalafil and sildenafil was headache [38, 39].

## Discussion

In this systematic review, we analyzed the evidence for interventional trials in RDs in rheumatology. Only a limited number of studies were identified. 50 trials fulfilled the inclusion criteria and were included in this systematic review (Fig. 1). Patient numbers and outcome measures varied across studies. Our study thus showed a broad heterogeneity of evidence for therapeutic regimens for the six diseases we were able to analyze.

Two RCTs which analyzed idursulfase against placebo [12, 13] in patients with Hunter syndrome demonstrated a significant result in all study outcome parameters. The inclusion criteria were stringent with a homogeneous patient population, and an acceptable total patient number (n = 85) (Fig. 4).

Of four tested regimens in Behçet’s disease [8, 15–18], two studies with apremilast [8, 18] and one with IF-α [15] were superior to placebo when looking at total or partial remission, with similar odds ratios (Fig. 5a, b). Of these two regimens, the apremilast studies of Hatemi et al. [8, 18] included more patients, and had a narrower CI and superior *p* value compared to the IF-a study (< 0.0001 vs. 0.31) with respect to complete remission. However, apremilast did not show a significant reduction of oral ulcerations, nor did any other regimen (Fig. 5c).

For giant cell arteritis, only Tocilizumab [7] showed a high degree of evidence with regard to relapse free remission (Fig. 6a). Other pharmacotherapies were less convincing.

In AAV, the results demonstrated a non-inferiority of RTX as compared to azathioprine and CYC [27–29]. Mepolizumab versus placebo [26] showed a significant increase in complete remission as primary outcome (Fig. 7).

None of the trials for reactive arthritis yielded favorable results in terms of change of CRP and swollen joint count [31–33], and only one study dates back less than 15 years (Fig. 8a,b). Nonetheless, comparing the patient’s global assessment, sulfasalazine [31] and a combination of rifampin and azithromycin [33] showed a significant effect (Fig. 8c).

Trials for Systemic Sclerosis focused on severe symptoms such as Raynaud’s phenomenon (Fig. 9b–d), pulmonary (Fig. 9a), and skin involvement (Fig. 9e). Iloprost [35] and bosentan [37] as compared to placebo resulted in significant improvement with regard to DLCO after treatment; however, the iloprost trial included only 30 patients (Fig. 9a). Thus, a higher number of patients would be necessary to confirm these results. A high-quality trial with nintedanib focused on the annual rate of decline in FVC (outcome was not improved upon treatment [42]), and not the DLCO, so it could not be included in the meta-analysis together with iloprost and bosentan. In addition, effects of iloprost, tadalafil and sildenafil on RP were limited. Improvement of skin involvement was only significant with CYC but not relaxin or nintedanib (Fig. 9e).

As outlined in the "Methods" section, we encountered problems with a more specific search strategy following the usual recommendations for systematic reviews, as we did not retrieve all relevant studies in this first attempt. In our view, it is therefore worth discussing whether the commonly recommended and used search strategy for systematic reviews actually applies to rare diseases. We therefore decided unanimously to slightly change our first strategy to find all relevant studies and exclude irrelevant studies later in the process according to our criteria (as outlined in the "Methods" section; search strategies can be found in Additional file 1: S2). The resulting promising RCTs are listed in Additional file 1: S3. However, not all of them could be included in the meta-analysis because of heterogeneities of outcome parameters.

In addition to the data represented in the meta-analysis, some high-quality individual studies demonstrated promising results. For example, for the periodic fever syndrome cryopyrine associated periodic syndrome (CAPS) we identified two studies which tested different interleukin-1 inhibitors (canakinumab and rilonacept), which showed a significant effect on disease activity [43, 44], but were not compatible with respect to outcome measures.

A number of studies had to be excluded because they had not enough participants. For example, we identified several relevant trials for another periodic fever syndrome, familial mediterranean fever (FMF). However, according to our criteria we could not include them because of the small number of patients. Similarly, exclusion because of too small participant numbers, as well as the exclusion of case series or case studies, had to be applied to a number of identified studies. Most likely this is due to the extreme rareness of some of the diseases of interest. For example, Muckle-Wells-Syndrome (ORPHA:575), which is characterized by chronic urticaria, arthritis, and fever, has an estimated prevalence of 1–10 cases per million [45].

Another high-quality RCT that could not be included in our meta-analysis due to incomparable outcome assessment is the RAPIDS-2 trial [46]. This study showed a significant reduction of digital ulcers after treatment with bosentan in patients with systemic sclerosis. Bosentan was subsequently licensed for this condition. In our systemic review of the literature we also found a study testing anakinra in Adult Onset Still’s Disease [47] which is a therapeutic alternative recently approved by the European Medicines Agency (EMA).

In contrast, many other studies such as high-dose immunoglobulins in sporadic inclusion body myositis [48] did not show a significant effect.

As mentioned above, heterogeneity of outcome measures limited our analysis and allowed only 26 out of 49 studies to be included. While sometimes a number of studies of the same disease were identified, for example systemic sclerosis, they often focused on specific symptoms, such as skin involvement, Raynaud’s phenomenon, digital ulcers, or pulmonary diffusing capacity, and the incomparability of outcome measures prevented inclusion in the meta-analysis. For example, two studies analyzing Raynaud’s phenomenon and interstitial lung disease could not be included here, because they tested different manifestations of the same disease. This made it difficult to compare the efficacy of interventions, which is especially regrettable in case of rare diseases with limited numbers of available candidates to participate in a study. Per definition, patient numbers in RDs are small, which hampers the design of high-quality RCTs.

In a previous systematic review concerning the issue of evidence-based clinical practice for rare diseases, Rath et al. [49] concluded that, as far as rare diseases are concerned, clinical data should be collected in databases and registries and more appropriate study designs adapted to small study populations should be selected. Especially in terms of (international) databases and transfer of knowledge, the situation has improved over the years, for example because of the European reference networks (ERN). In addition to these networks, other organizations supported this development such as the European Scleroderma Trials and Research group (EUSTAR) who built a multicenter online database. Nonetheless further efforts are needed.

One strategy to overcome the difficulty of the scarcity of patients has been employed by Stone et al. [28], who have pooled different but related diseases such as GPA and MPA. This resulted in a greatly elevated number of 197 study patients with AAV patients. On the other hand, this leads to less consistent groups of participants compared to studies which focused only on one defined disease, like Wechsler et al. [26] who only included patients with EGPA. The limitations caused by low patient numbers were particularly obvious for ultra-rare diseases, none of which could be included in our analysis since there were no RCTs meeting the criteria, as case studies and trials with less than 10 participants per arm were excluded.

Lastly, limitations might have arisen from the fact that our literature research concentrated on four major databases, but we still estimate that it most likely covered a majority of data available.

## Conclusion

Patients with RDs are rare by definition and often do not show the entire spectrum of symptoms, which frequently results in a long time until diagnosis. Concerning our objectives to identify RCTs dealing with RDs in rheumatology, evaluate study quality on the basis of risk of bias, and elucidate the findings from pharmacotherapeutic RCTs, we can summarize that there are several randomized controlled trials, even with high quality in terms of risk of bias. Most of the trials included in our meta-analysis demonstrated an improvement when tested against placebo or other standard therapies with an overall satisfactory safety. Since many RDs in rheumatology lack randomized controlled trials and treatment guidelines, therapeutic strategies are often based only on case studies or clinical experience, further contributing to the disadvantages of patients with RDs. This problem is further exacerbated by a lack of standardized outcome measures in the design of the studies. Our meta-analysis may help to shed light on these issues in this field of medicine. It may also encourage physicians to more often consider a RD as a differential diagnosis with a limited set of therapeutic options. At the same time, more RCTs are urgently needed to cover this great unmet need.

## Supplementary information

**Additional file 1.** Applied search strategy for each database.

## Data Availability

All data discussed are included with the published article and the supplementary table.

## Abbreviations

AAV: ANCA-associated vasculitis
CAPS: Cryopyrin associated periodic syndrome
CI: Confidence interval
CRP: C-reactive protein
CYC: Cyclophosphamide
DLCO: Diffusing capacity or transfer factor of the lung for carbon monoxide
DMARD: Disease modifying antirheumatic drug
GAG: Glycosaminoglycan
GCA: Giant cell arteritis
GPA: Granulomatosis with polyangiitis
ERN: European reference network
EUSTAR: European scleroderma trials and research group
EULAR: European league against rheumatism
EGPA: Eosinophilic granulomatosis with polyangiitis
EMA: European medicines agency
FVC: Functional vital capacity
mRSS: Modified Rodnan Skin Score
MPA: Microscopic polyangiitis
MTX: Methotrexate
RCT: Randomized controlled trial
ReA: Reactive arthritis
RD: Rare disease
RP: Raynaud’s phenomenon
SSC: Systemic sclerosis
