# Supplementary material for: Therapeutic options for patients with rare rheumatic diseases: a systematic review and meta-analysis
Source: Orphanet J Rare Dis. 2020 Oct 31;15:308. doi: 10.1186/s13023-020-01576-5 (PMC7603763; doi:10.1186/s13023-020-01576-5)
Supplement: Supplementary file 1 — Additional file 1. Applied search strategy for each database. [file 13023_2020_1576_MOESM1_ESM.docx]

| S1: Table of included studies | | | | | | |
| --- | --- | --- | --- | --- | --- | --- |
| **Study** | **Design** | **Disease** | **Therapy** | **Number of Patients** | **Setting** | **Outcome measures** |
| Alpsoy, 2002 | randomized, placebo-controlled and double-blind trial | Behçet‘s disease | interferon alpha | 44 | a. interferon alpha-2a 6 x 10^6 IU, 3/week  b. placebo, 3/week | Frequency, duration and pain of oral and genital ulcers: assessed  Frequency and duration of articular symptoms: assessed  Ocular manifestations: assessed  Erythema nodosum: assessed |
| Andrigueti, 2017 | randomized, double-blinded, placebo-controlled trial | Systemic sclerosis | sildenafil | 41 | a. sildenafil  b. placebo | changes in the finger blood flow  serum levels of EPCs, VEGF, frequency and duration of Raynaud Phenomenon (RP) attacks, visual analog scale (VAS) score for RP severity, and the Raynaud’s Condition Score. |
| Au, 2010 | double-blind, randomized, placebo-controlled trial | Systemic sclerosis | Cyclophosphamide (CYC) | 158 | a. CYC  b. placebo | digital tip ulcers defined as ulcers distal to the distal interphalangeal joint;  other dermal ulcers  joint swelling  joint tenderness at the wrists, elbows, knees, and MCP joints  muscle tenderness on palpation documented as either present or absent  modified Rodnan skin thickness score  pulmonary function tests, including FVC, DLCO, maximal inspiratory pressures (MIPs), and maximal expiratory pressures (MEPs)  the disability index (DI) of the Health Assessment Questionnaire (HAQ)  the Medical Outcomes Survey Short Form 36 (SF-36) |
| Banikazemi 2007 | Randomized (2:1 treatment-to-placebo randomization), double-blind, placebo-controlled trial | Fabry's disease | agalsidase beta | 82 | a. agalsidase beta  b. placebo | time to first clinical event (renal, cardiac, or cerebrovascular event or death).  The following measures were performed or obtained at baseline and at the final study visit or time of study withdrawal: serum creatinine level, proteinuria (ratio of urinary protein to urinary creatinine [in mg/dL]), ratio of urinary albumin to urinary creatinine (in mg/dL), 12-lead electrocardiography, echocardiography, neurologic examination, head magnetic resonance imaging, Brief Pain Inventory, exercise tolerance, plasma globotriaosylceramide level, Fabry symptom assessment, physical examination, blood chemistries, urinalysis, IgG antibody titers to agalsidase beta, and optional skin biopsy. |
| Calgüneri, 1996 | prospective randomized, parallel design study | Behçet‘s disease | colchicine + benzathine penicillin | 120 | a. colchicine + benzathine penicillin  b. colchicine | The incidence of arthritis episodes during a period of 24 months of therapy |
| Carter, 2010 | double-blind, triple-dummy prospective trial | Chlamydia-induced reactive Arthritis | combination antibiotics | 42 | a. doxycycline 100mg per-oral twice daily and rifampin 300mg per-oral daily plus azithromycin placebo  b. azithromycin 500mg per-oral daily for 5 days and then 500mg per-oral twice weekly and rifampin 300mg per-oral daily plus doxycycline placebo  c. azithromycin, doxycycline, and rifampin placebos | Remission rate; CRP; ESR |
| Clegg, 1996 | double-blind, placebo controlled, randomized trial | reactive arthritis | Sulfasalazin | 134 | a. sulfasalazin 2000mg/day  b. placebo | Patient and clinical assessment; Laboratory assessment; radiology assessment |
| Cohen, 2007 | prospective, multicenter, randomized controlled trial | Churg-Strauss-Syndrome | CYC | 48 | a. 6-pulse CYC  b. 12-pulse CYC | Birmingham Vaskulitis Activity Score (BVAS): assessed  complete remission: assessed |
| Distler, 2019 | randomized, double-blind, placebo-controlled, parallel-group trial | Systemic sclerosis | Nintedanib | 576 | a. 150 mg of nintedanib  b. Placebo | annual rate of decline in forced vital capacity (FVC)  absolute changes from baseline in the modified Rodnan skin score  absolute change from baseline in the total score on the St. George’s Respiratory Questionnaire |
| Hachulla, 2016 | prospective, longitudinal, randomised, comparative, double-blind, two-parallel-arm, placebo-controlled trial | Systemic sclerosis | sildenafil | 84 | a. sildenafil  b. placebo | time to healing of ischemic digital ulcers (DUs) |
| Han, 2011 | prospective, randomized, open-label trial | microscopc polyangiitis | mycophenolat mofetil, CYC | 41 | a. mycophenolate mofetil (mmf) 1g/d  b. i.v. CYC 1g/month + corticosteroids  c. both groups methylprednisolone 360-500mg/day for 3 days + orl prednisone 0,6-0,8 mg/kg/d | eGFR: assessed  complete remission after 6 months: assessed by BVAS  adverse events rate: assessed |
| Hatemi, 2015 | double-blind, placebo-controlled, parallel-group study | Behçet’s disease | apremilast | 111 | a. apremilast  b. placebo | 1) number of oral ulcers  2) change in pain of oral and genital ulcers from baseline to 12 weeks  3) Behçet’s Disease Current Activity Form  4) Behçet’s Syndrome Activity Score  5) Behçet’s Disease Quality of Life scale  6) SF-36 |
| Hatemi, 2019 | double-blind, placebo-controlled, parallel-group study | Behçet’s disease | apremilast | 207 | a. apremilast  b. placebo | 1) area under the curve (AUC) for the total number of oral ulcers  2) change in pain of oral and genital ulcers from baseline to 12 weeks  3) Behçet’s Disease Current Activity Form  4) percentage of patients free from oral ulcers by week 6 who remained oral ulcer–free for at least 6 weeks  5) change from baseline in the Behçet’s Disease Quality of Life score |
| Hoffman, 2002 | randomized, double-blind, placebo-controlled trial | Giant cell arteriitis | MTX | 98 | a. Corticosteroids (CS) + Methotrexate (MTX)  b. CS + placebo | number of disease relapses and treatment failures in the two groups |
| Hoffman, 2007 | randomized, double-blind, placebo-controlled trial | Giant cell arteriitis | infliximab | 44 | a. CS + infliximab 5mg/kg  b. CS + placebo | 1)Primary end point: number of patients who remained free of a relapse and adverse events until week 22  2)Secondary end points: time to first relapse, cumulative glucocorticosteroid dose, and the number of patients who remained relapse-free while the glucocorticosteroid dosage was tapered to 10 mg/d |
| Hoffmann, 2008 | two sequential blinded and placebo-controlled trials | CAPS | rilonacept | 47 | a. rilonacept 160mg s.c.  b. placebo | change in the mean key symptom score derived from the daily health assessment form (DHAF) as compared to the baseline evaluation period (3-week period prior to randomization) to the end point evaluation period (last 3 weeks of double-blind treatment) |
| Jones, 2010 | open-label, two-group, parallel-design, randomized trial | ANCA-associated vasculitis | Rituximab (RTX) | 44 | a. RTX 375 mg per square meter per week + CYC 15mg/kg  b. CYC 15 mg/kg | sustained remission rates and severe adverse event at 12 months. Remission was defined as an absence of clinical disease activity, as indicated by a Birmingham Vasculitis Activity Score (BVAS) of 0 that was maintained for 2 months (scores range from 0 to 63, with higher scores indicating more active disease). Sustained remission was defined as an absence of disease activity (BVAS of 0) for at least 6 months |
| Jover, 2001 | randomized, double-blind, placebo-controlled trial | Giant cell arteriitis | MTX | 42 | a. CS + MTX  b. CS + placebo | Number of relapses, cumulative dose of corticosteroid, and number of adverse events were assessed on completion of follow-up. |
| Khanna, 2009 | Randomized controlled trial | Systemic Sclerosis | relaxin | 231 | a. relaxin 10µg/kg/d  b. relaxin 25µg/kg/d  c. placebo | The primary measure of efficacy was the MRSS, a clinical evaluation of skin thickness in 17 body surface areas (face, chest, and abdomen, and right and left fingers, hands, forearms, upper arms, thighs, lower legs, and feet) |
| Khanna, 2016 | randomized, double-blind, placebo-controlled trials | Systemic Sclerosis | macitentan | 299 | a. macitentan 3mg  b. macitentan 10mg  c. placebo | cumulative number of new digital ulcers from baseline to week 16 |
| Kilic, 2009 | randomized, placebo controlled, double blind study | Behçet’s disease | Interferon apha (IFN alpha) | 84 | a. 2000 IU IFN alpha  b. placebo  c. 1000 IU IFN alpha  1) primary | changes from baseline in the total oral ulcer burden in each patient. |
| Korn, 2004 | randomized, double-blind, placebo-controlled trial | Systemic Sclerosis | bosentan | 122 | a. bosentan 62,5mg 2x/d  b. Placebo | 1)primary outcome parameter number of new digital ulcers developing during the 16-week study treatment period.  2)Secondary a priori outcome variables included time to complete or partial healing of baseline digital ulcers, time to onset of new ulcers, and SHAQ subsets for dressing, hygiene, grip, and hand function. In addition, assessment of safety and tolerability. |
| Köse, 2009 | randomized, double-blind, controlled trial | Behçet’s disease | Pimecrolimus + Colchizin | 68 | a. pimecrolimus cream plus colchicine tablets  b. placebo | Mean healing time of ulcers. Also, genital ulcer pain was evaluated using a verbal pain score at each visit. Safety was monitored through adverse event reporting and laboratory tests. |
| Lachmann, 2009 | randomized, double-blind, controlled trial | CAPS | canakinumab | 31 | a. canakinumab  b. placebo | proportion of patients with a relapse of CAPS during canakinumab treatment, as compared with placebo.  proportion of patients with a complete response in part 1, values of inflammatory markers, global assessments by physicians and patients, and safety and tolerability. |
| Lightman, 2015 | randomized, controlled, parallel group, single-masked clinical trial | Behçet’s disease | peginterferon-alpha-2b | 72 | a. peginterferon-α-2b + the patient's standard therapy  b. the patient's standard care | The primary outcome measure was whether a corticosteroid dose equivalent to no more than 10 mg of prednisolone per day was required throughout months 10–12 following treatment initiation.  Secondary endpoints:  number of disease relapses, mean doses of corticosteroids and immunosuppressive agents required for disease control, and questionnaire scores up to 3 years. |
| Mat, 2006 | randomized, placebo-controlled study | Behçet’s disease | depot corticosteroid injections | 86 | a. depot corticosteroid intramuscular injections (40 mg methylprednisolone acetate)  b. placebo | difference in the mean number of genital ulcers  differences in the mean numbers of other mucocutaneous lesions and attacks of arthritis |
| Matucci-Cerinic, 2011 | double-blind, placebo-controlled trial | Systemic Sclerosis | bosentan | 188 | a. bosentan  b. placebo | (1) mean number of new DUs per patient assessed by the investigator up to week 24 and  (2) time to healing of the cardinal ulcer up to week 24 in patients with cardinal ulcer healing maintained for 12 or more weeks. |
| Mazlumzadeh, 2006 | double-blind, placebo-controlled, randomized prospective clinical trial | Giant cell arteriitis | methylprednisolone | 28 | a. pulse IV methylprednisolone 15 mg/kg  b. IV normal saline as placebo | The primary outcome measure was met if a patient was taking ≤5 mg/day of oral prednisone 36 weeks after the initiation of GC therapy. |
| Milio, 2006 | randomized, prospective, controlled, open study with parallel groups | Systemic Sclerosis | iloprost | 60 | a. iloprost at progressively increasing doses (from 0.5 to 2 ng/kg/min) for 6 h a day. The treatment proceeded for 10 days for two consecutive weeks (5 consecutive days + 2 days of interruption + 5 consecutive days) with repeated cycles four times a year at regular intervals of 3 months. (Group A)  b. iloprost infusin 1/month (Group B1)  c. iloprost 20 consecutive infusions every 6 months (Group B2) | duration, number and severity of Raynaud's attacks |
| Miller, 1992 | double-blind, placebo-controlled trial | Polymyositis/Dermatomyositis | plasma exchange, leukapheresis | 39 | a. plasma exchange  b. leukapheresis  c. sham apharesis | changes in strength and functional levels. |
| Muenzer, 2006 | randomized, double-blind, placebo-controlled, clinical trial | Hunter syndrome | idursulfase | 96 | a. idursulfase 0.5 mg/kg weekly  b. idursulfase 0.5 mg/kg every-other-week  c. placebo | %FVC as a measure of respiratory function  6MWT as a measure of physical functional capacity. |
| Nordström, 2012 | open, randomized (1:1), multicenter trial | Adult Onset Still’s Disease (AOSD) | anakinra | 22 | a. anakinra  b. DMARD | Remission. Tender Joint Count (TJC), Swollen Joint Count (SJC) |
| Oddis, 2013 | randomized, placebo-phase design (RPPD) | Polymyositis/Dermatomyositis | rituximab | 200 | a. rituximab  b. placebo | The primary end point was the time to achieve the DOI, which was compared between the rituximab early and rituximab late groups. |
| Putschky, 2006 | prospective, randoised, double-blind, controlled clinical trial | Reactive Arthritis | doxycyclin | 32 | a. doxycycline 4 months  b. doxycyclin 10 days + placebo | Patient's global assessment and intensity of pain (visual analogue scales), as well as duration of morning stiffness and fatigue (measured in min). The parameters collected by clinical examination were the number of tender joints and number of swollen joints.  At the beginning and at the end of the trial, erythrocyte sedimentation rate (ESR), C reactive protein level were measured. |
| Ribi, 2008 | prospective, randomized, open-label trial | Churg-Strauss-Syndrome | CS, azathiprine, CYC | 72 | a. corticosteroids alone (n=53)  b. azathioprine 2mg/kg/d for 6 months (n=9)  c. cyclophosphamide 600mg/m2 every 2 weeks for 1 month, then every 4 weeks (n=10) | Complete remission. |
| Schiffmann, 2001 | Double-blind placebo-controlled trial | Fabry's disease | alpha-galactosidase a | 26 | a. alpha-galactosidase a  b. placebo | The primary efficacy end point was the effect of therapy on neuropathic pain while without pain medications, as measured by the "pain at its worst" item (question 3) from the BPI.  At baseline and at week 24, inulin clearance and creatinine clearance were used to estimate glomerular filtration rate, and renal biopsies were performed. |
| Schiopu, 2009 | prospective, randomized, double-blind, placebo-controlled, crossover study | Systemic Sclerosis | tadalafil | 39 | a. tadalafil  b. placebo | Raynaud Condition Score (RCS), frequency of RP episodes, or duration of RP |
| Scorza, 2001 | prospective, randoised, double-blind, controlled clinical trial | Systemic Sclerosis | iloprost, Nifedipin | 46 | a. iloprost  b. nifedipin | Modified Rodnan skin score (mRSS), pulmonary function and Raynaud’s severity score in patients with SSc and RP. |
| Seibold, 2010 | prospective, double-blind, randomized, placebo-controlled, parallel group trial | Systemic Sclerosis | bosentan | 152 | a. bosentan  b. placebo | The primary end point for this study was a change in the 6-minute walk distance from baseline up to month 12. |
| Seror, 2014 | randomized, double-blind, placebo controlled trial | Giant cell arteritis | adalimumab | 70 | a. adalimumab 40mg  b. placebo | The primary endpoint was the percentage of patients in remission with less than 0.1 mg/kg per day of prednisone at week 26. The secondary objectives were to assess the decrease in prednisone dose during the first 6 months of treatment, the proportion of patients who remained relapse free at 1 year, and the safety of a 10-week treatment with adalimumab in patients with GCA. |
| Sieper, 1999 | double-blind, randomized, placebo controlled study | Reactive Arthritis | ciprofloxacin | 55 | a. ciprofloxacin  b. placebo | The percentage of patients in remission after 3 months of treatment was chosen as the primary efficacy parameter. |
| Sohn, 2013 | randomized, single-blinded, active comparator-controlled clinical trial | Hunter Syndrome | idursulfase beta | 31 | a. idursulfase beta group, 0.5 mg/kg/week  b. idursulfase beta group, 1.0 mg/kg/week  c. comparator | The primary endpoint was the extent of reduction in urinary GAG excretion. Urinary GAG concentrations were quantified by cerylpyridinium chloride (CPC) precipitation, normalized to urinary creatinine concentration, and reported as CPC unit/g creatinine. |
| Specks, 2013 | randomized, double-blind, double-dummy, noninferiority trial | ANCA-positive vasculitis | rituximab | 197 | a. rituximab 375 mg per square meter of body-surface area per week for 4 weeks + CYC Placebo + methylprednisolone  b. CYC 2 mg per kilogram of body weight per day + Rituximab Placebo + methylprednisolone | The primary comparison at 12 and 18 months was the percentage of patients who had a score of 0 on the BVAS/WG, had completed the glucocorticoid-tapering regimen, and had not had a relapse or any other reason for treatment failure before the time point of interest. Adverse events were recorded for all patients from the time of enrollment through the closeout date of the study. BVAS, PGA, SF-36 and VDI were assessed. |
| Stone, 2010 | randomized, double-blind, double-dummy, noninferiority trial | ANCA-positive vasculitis | rituximab | 197 | a. rituximab 375 mg per square meter of body-surface area per week for 4 weeks + CYC Placebo + methylprednisolone  b. CYC 2 mg per kilogram of body weight per day + Rituximab Placebo + methylprednisolone | The primary end point was a BVAS/WG of 0 and successful completion of the prednisone taper at 6 months. Secondary end points included rates of disease flares, a BVAS/WG of 0 during treatment with prednisone at a dose of less than 10 mg per day, cumulative glucocorticoid doses, rates of adverse events, and SF-36 scores. BVAS, PGA, SF-36 and VDI were assessed. |
| Stone, 2017 | The trial consists of a 52-week blinded period (Part 1), followed by a 104-week open-label extension (Part 2) | Giant cell arteritis | tocilizumab | 200 | a. TCZ 162qw + 26 weeks of prednisone + 26 weeks of prednisone placebo  b. TCZ 162q2w + 26 weeks of prednisone + 26 weeks of prednisone placebo  c. TCZ-placebo + 26 weeks of prednisone + 26 weeks of prednisone placebo  d. TCZ-placebo + 52 weeks of prednisone | The primary outcome was the rate of sustained glucocorticoid-free remission at week 52 in each tocilizumab group as compared with the rate in the placebo group that underwent the 26-week prednisone taper. The key secondary outcome was the rate of remission in each tocilizumab group as compared with the placebo group that underwent the 52-week prednisone taper. Dosing of prednisone and safety were also assessed. |
| Tashkin, 2006 | double-blind, randomized, placebo-controlled trial | Systemic Sclerosis | CYC | 158 | a. CYC  b. placebo | 1.Spirometry. 2.Whole-body plethysmographic lung volumes. 3.DLCO and ratio of DLCO to alveolar volume (VA). 4.Maximum inspiratory and expiratory mouth pressures. 5.Modified Rodnan skin thickness score (0-51, high score being worse). 6.BDI (total score 0-12, low score being worse). 7.Modified cough index (severity: 0-3, high score being worse). 8.The 36-item Medical Outcomes Survey (MOS-SF36) . 9.20-item Health Assessment Questionnaire-Disability Index (HAQ-DI) modified for scleroderma (0-3, high score being worse) |
| van de Vlekkert, 2010 | double-blind randomised controlled clinical trial | inflammatory myopathies | dexamethasone | 62 | a. 28-day cycles of oral high-dose dexamethasone  b. daily high-dose prednisolone | There were two primary outcome measures: (1) a composite score of measures for favorable outcome at 18 months of followup or at the time point of discontinuation of the trial; (2) (time to) remission and relapse. |
| Walter, 2000 | randomized, double-blind, placebo-controlled, cross-over study | Inclusion body Myositis (IBM) | Intravenous Immunglobuline (IVIG) | 22 | a. 2 g/kg intravenous immunoglobulin therapy (IVIG) monthly  b. placebo | After 6 and 12 months the response to treatment was evaluated, using a modified Medical Research Council scale (MRC) for muscle strength, Neuromuscular Symptom Score (NSS), the patient's own assessment of improvement, arm outstretched time, and electromyography. |
| Wechsler, 2017 | randomized, placebo-controlled, double-blind, parallel-group trial | Eosinophilic Granulomatosis with Polyangiitis | mepolizumab | 136 | a. mepolizumab  b. placebo | The first primary end point was the total accrued weeks of remission. Remission was defined as a Birmingham Vasculitis Activity Score (BVAS), version 3, of 0 (on a scale from 0 to 63, with higher scores indicating greater disease activity) and the receipt of prednisolone or prednisone at a dose of 4.0 mg or less per day over the 52-week period |
| Yurdakul, 2001 | randomized, double blind prospective trial | Behcet's disease | colchicine | 116 | a. colchicine  b. placebo | The effects of colchicine or placebo on each lesion were analyzed separately. |
|  |  |  |  |  |  |  |

**S2: Search Strategies**

Pubmed

| #1 | Title | „microscopic polyangiitis” OR “systemic sclerosis” OR “polimyositis” OR “dermatomyositis” OR “churg-strauss syndrome” OR "Behçet syndrome" OR “cryglobulinemia” OR “Mediterranean fever” OR “giant cell arteritis” OR “Wegener granulomatosis” OR “granulomatosis with polyangiitis” OR “hunter syndrome” OR “reiter syndrome” OR “muckle-wells syndrome” OR “still’s disease” OR “fabry disease” OR “McArdle disease” OR “Antisynthetase syndrome” OR “ehlers danlos syndrome” OR “tumor necrosis factor receptor-associated periodic syndrome” |
| --- | --- | --- |
| #2 | Title/Abstract | Therapy OR treatment |
| #3 | Publication type | „Randomized controlled trial“ |
| #4 |  | #1 AND #2 AND #3 |

CENTRAL

| #1 | "systemic sclerosis":ti or "microscopic polyangiitis":ti or "polymyositis":ti or "dermatomyositis":ti |
| --- | --- |
| #2 | "Churg-Strauss syndrome":ti or "Behçet syndrome":ti or "cryglobulinemia":ti or "Mediterranean fever":ti or "giant cell arteritis":ti |
| #3 | "Wegener granulomatosis":ti or "granulomatosis with polyangiitis":ti or "Hunter syndrome":ti or "Reiter syndrome":ti or "Muckle-Wells syndrome":ti |
| #4 | "Still's disease":ti or "Fabry disease":ti or "McArdle disease":ti or "antisynthetase syndrome":ti or "Ehlers Danlos syndrome":ti |
| #5 | "tumor necrosis factor receptor-associated periodic syndrome":ti,ab,kw |
| #6 | "therapy":ti,ab,kw or "treatment":ti,ab,kw |
| #7 | "randomised controlled trial":pt or "randomized controlled trial":pt in Trials (Word variations have been searched) |
| #8 | #1 or #2 or #3 or #4 or #5 and #6 and #7 |
|  |  |

MEDLINE / EMBASE

| #1 | Title | microscopic polyangiitis OR systemic sclerosis OR polimyositis OR dermatomyositis OR churg-strauss syndrome OR Behcet syndrome OR cryglobulinemia OR Mediterranean fever OR giant cell arteritis OR Wegener granulomatosis OR granulomatosis with polyangiitis OR hunter syndrome OR reiter syndrome OR muckle-wells syndrome OR still disease OR fabry disease OR McArdle disease OR Antisynthetase syndrome OR ehlers danlos syndrome OR tumor necrosis factor receptor-associated periodic syndrome |
| --- | --- | --- |
| #2 | Title/Abstract | Therapy OR treatment |
| #3 | Publication type | Randomized controlled trial |

**S3 Bibliography of all studies included in the systematic review**

1. Alpsoy E, Durusoy C, Yilmaz E, Ozgurel Y, Ermis O, Yazar S, u. a. Interferon alfa-2a in the treatment of Behcet disease: a randomized placebo-controlled and double-blind study. Arch Dermatol. April 2002;138(4):467–71.

2. Andrigueti FV, Ebbing PCC, Arismendi MI, Kayser C. Evaluation of the effect of sildenafil on the microvascular blood flow in patients with systemic sclerosis: a randomised, double-blind, placebo-controlled study. Clin Exp Rheumatol. Oktober 2017;35 Suppl 106(4):151–8.

3. Au K, Mayes MD, Maranian P, Clements PJ, Khanna D, Steen VD, u. a. Course of dermal ulcers and musculoskeletal involvement in systemic sclerosis patients in the scleroderma lung study. Arthritis Care Res. Dezember 2010;62(12):1772–8.

4. Banikazemi M, Bultas J, Waldek S, Wilcox WR, Whitley CB, McDonald M, u. a. Agalsidase-beta therapy for advanced Fabry disease: a randomized trial. Ann Intern Med. 16. Januar 2007;146(2):77–86.

5. Calgüneri M, Ertenli I, Kiraz S, Erman M, Celik I. Effect of prophylactic benzathine penicillin on mucocutaneous symptoms of Behçet’s disease. Dermatol Basel Switz. 1996;192(2):125–8.

6. Carter J, Espinoza L, Inman R, Sneed K, Ricca L, Vasey F, u. a. Combination antibiotics as a treatment for chronic Chlamydia-induced reactive arthritis: a double-blind, placebo-controlled, prospective trial. Arthritis Rheum. 2010;62(5):1298–307.

7. Clegg DO, Reda DJ, Weisman MH, Cush JJ, Vasey FB, Schumacher HRJ, u. a. Comparison of sulfasalazine and placebo in the treatment of reactive arthritis (Reiter’s syndrome). A Department of Veterans Affairs Cooperative Study. Arthritis Rheum. 1996;39(12):2021–7.

8. Cohen P, Pagnoux C, Mahr A, Arene J-P, Mouthon L, Le Guern V, u. a. Churg-Strauss syndrome with poor-prognosis factors: A prospective multicenter trial comparing glucocorticoids and six or twelve cyclophosphamide pulses in forty-eight patients. Arthritis Rheum. 15. Mai 2007;57(4):686–93.

9. Hachulla E, Hatron P-Y, Carpentier P, Agard C, Chatelus E, Jego P, u. a. Efficacy of sildenafil on ischaemic digital ulcer healing in systemic sclerosis: the placebo-controlled SEDUCE study. Ann Rheum Dis. Juni 2016;75(6):1009–15.

10. Han F, Liu G, Zhang X, Li X, He Q, He X, u. a. Effects of mycophenolate mofetil combined with corticosteroids for induction therapy of microscopic polyangiitis. Am J Nephrol. 2011;33(2):185–92.

11. Hatemi G, Melikoglu M, Tunc R, Korkmaz C, Turgut Ozturk B, Mat C, u. a. Apremilast for Behçet’s syndrome--a phase 2, placebo-controlled study. N Engl J Med. 2015;372(16):1510–8.

12. Hoffman GS, Cid MC, Rendt-Zagar KE, Merkel PA, Weyand CM, Stone JH, u. a. Infliximab for maintenance of glucocorticosteroid-induced remission of giant cell arteritis: a randomized trial. Ann Intern Med. 2007;146(9):621–30.

13. Hoffman GS, Cid MC, Hellmann DB, Guillevin L, Stone JH, Schousboe J, u. a. A multicenter, randomized, double-blind, placebo-controlled trial of adjuvant methotrexate treatment for giant cell arteritis. Arthritis Rheum. Mai 2002;46(5):1309–18.

14. Hoffman HM, Throne ML, Amar NJ, Sebai M, Kivitz AJ, Kavanaugh A, u. a. Efficacy and safety of rilonacept (interleukin-1 Trap) in patients with cryopyrin-associated periodic syndromes: results from two sequential placebo-controlled studies. Arthritis Rheum. August 2008;58(8):2443–52.

15. Jones RB, Tervaert JWC, Hauser T, Luqmani R, Morgan MD, Peh CA, u. a. Rituximab versus cyclophosphamide in ANCA-associated renal vasculitis. N Engl J Med. 15. Juli 2010;363(3):211–20.

16. Jover JA, Hernandez-Garcia C, Morado IC, Vargas E, Banares A, Fernandez-Gutierrez B. Combined treatment of giant-cell arteritis with methotrexate and prednisone. a randomized, double-blind, placebo-controlled trial. Ann Intern Med. 16. Januar 2001;134(2):106–14.

17. Khanna D, Clements PJ, Furst DE, Korn JH, Ellman M, Rothfield N, u. a. Recombinant human relaxin in the treatment of systemic sclerosis with diffuse cutaneous involvement: a randomized, double-blind, placebo-controlled trial. Arthritis Rheum. April 2009;60(4):1102–11.

18. Khanna D, Denton CP, Merkel PA, Krieg T, Le Brun F-O, Marr A, u. a. Effect of Macitentan on the Development of New Ischemic Digital Ulcers in Patients With Systemic Sclerosis: DUAL-1 and DUAL-2 Randomized Clinical Trials. JAMA. 10. Mai 2016;315(18):1975–88.

19. Kiliç H, Zeytin HE, Korkmaz C, Mat C, Gül A, Coşan F, u. a. Low-dose natural human interferon-alpha lozenges in the treatment of Behçet’s syndrome. Rheumatol Oxf Engl. November 2009;48(11):1388–91.

20. Korn J, Mayes M, Matucci Cerinic M, Rainisio M, Pope J, Hachulla E, u. a. Digital ulcers in systemic sclerosis: prevention by treatment with bosentan, an oral endothelin receptor antagonist. Arthritis Rheum. 2004;50(12):3985–93.

21. Köse O, Dinç A, Simşek I. Randomized trial of pimecrolimus cream plus colchicine tablets versus colchicine tablets in the treatment of genital ulcers in Behçet’s disease. Dermatol Basel Switz. 2009;218(2):140–5.

22. Lachmann HJ, Kone-Paut I, Kuemmerle-Deschner JB, Leslie KS, Hachulla E, Quartier P, u. a. Use of Canakinumab in the Cryopyrin-Associated Periodic Syndrome. N Engl J Med. 4. Juni 2009;360(23):2416–25.

23. Lightman S, Taylor S, Bunce C, Longhurst H, Lynn W, Moots R, u. a. Pegylated interferon-α-2b reduces corticosteroid requirement in patients with Behçet’s disease with upregulation of circulating regulatory T cells and reduction of Th17. Ann Rheum Dis. 2015;74(6):1138–44.

24. Mat C, Yurdakul S, Uysal S, Gogus F, Ozyazgan Y, Uysal O, u. a. A double-blind trial of depot corticosteroids in Behçet’s syndrome. Rheumatol Oxf Engl. März 2006;45(3):348–52.

25. Matucci-Cerinic M, Denton CP, Furst DE, Mayes MD, Hsu VM, Carpentier P, u. a. Bosentan treatment of digital ulcers related to systemic sclerosis: results from the RAPIDS-2 randomised, double-blind, placebo-controlled trial. Ann Rheum Dis. Januar 2011;70(1):32–8.

26. Mazlumzadeh M, Hunder GG, Easley KA, Calamia KT, Matteson EL, Griffing WL, u. a. Treatment of giant cell arteritis using induction therapy with high-dose glucocorticoids: a double-blind, placebo-controlled, randomized prospective clinical trial. Arthritis Rheum. Oktober 2006;54(10):3310–8.

27. Milio G, Corrado E, Genova C, Amato C, Raimondi F, Almasio PL, u. a. Iloprost treatment in patients with Raynaud’s phenomenon secondary to systemic sclerosis and the quality of life: a new therapeutic protocol. Rheumatol Oxf Engl. August 2006;45(8):999–1004.

28. Miller FW, Leitman SF, Cronin ME, Hicks JE, Leff RL, Wesley R, u. a. Controlled trial of plasma exchange and leukapheresis in polymyositis and dermatomyositis. N Engl J Med. 21. Mai 1992;326(21):1380–4.

29. Muenzer J, Wraith JE, Beck M, Giugliani R, Harmatz P, Eng CM, u. a. A phase II/III clinical study of enzyme replacement therapy with idursulfase in mucopolysaccharidosis II (Hunter syndrome). Genet Med Off J Am Coll Med Genet. August 2006;8(8):465–73.

30. Nordström D, Knight A, Luukkainen R, van Vollenhoven R, Rantalaiho V, Kajalainen A, u. a. Beneficial effect of interleukin 1 inhibition with anakinra in adult-onset Still’s disease. An open, randomized, multicenter study. J Rheumatol. Oktober 2012;39(10):2008–11.

31. Oddis CV, Reed AM, Aggarwal R, Rider LG, Ascherman DP, Levesque MC, u. a. Rituximab in the treatment of refractory adult and juvenile dermatomyositis and adult polymyositis: a randomized, placebo-phase trial. Arthritis Rheum. Februar 2013;65(2):314–24.

32. Putschky N, Pott H, Kuipers J, Zeidler H, Hammer M, Wollenhaupt J. Comparing 10-day and 4-month doxycycline courses for treatment of Chlamydia trachomatis-reactive arthritis: a prospective, double-blind trial. Ann Rheum Dis. 2006;65(11):1521–4.

33. Ribi C, Cohen P, Pagnoux C, Mahr A, Arene J-P, Lauque D, u. a. Treatment of Churg-Strauss syndrome without poor-prognosis factors: a multicenter, prospective, randomized, open-label study of seventy-two patients. Arthritis Rheum. Februar 2008;58(2):586–94.

34. Schiffmann R, Kopp JB, Austin HA 3rd, Sabnis S, Moore DF, Weibel T, u. a. Enzyme replacement therapy in Fabry disease: a randomized controlled trial. JAMA. 6. Juni 2001;285(21):2743–9.

35. Schiopu E, Hsu VM, Impens AJ, Rothman JA, McCloskey DA, Wilson JE, u. a. Randomized placebo-controlled crossover trial of tadalafil in Raynaud’s phenomenon secondary to systemic sclerosis. J Rheumatol. Oktober 2009;36(10):2264–8.

36. Scorza R, Caronni M, Mascagni B, Berruti V, Bazzi S, Micallef E, u. a. Effects of long-term cyclic iloprost therapy in systemic sclerosis with Raynaud’s phenomenon. A randomized, controlled study. Clin Exp Rheumatol. Oktober 2001;19(5):503–8.

37. Seibold JR, Denton CP, Furst DE, Guillevin L, Rubin LJ, Wells A, u. a. Randomized, prospective, placebo-controlled trial of bosentan in interstitial lung disease secondary to systemic sclerosis. Arthritis Rheum. Juli 2010;62(7):2101–8.

38. Seror R, Baron G, Hachulla E, Debandt M, Larroche C, Puéchal X, u. a. Adalimumab for steroid sparing in patients with giant-cell arteritis: results of a multicentre randomised controlled trial. Ann Rheum Dis. 2014;73(12):2074–81.

39. Sieper J, Fendler C, Laitko S, Sörensen H, Gripenberg-Lerche C, Hiepe F, u. a. No benefit of long-term ciprofloxacin treatment in patients with reactive arthritis and undifferentiated oligoarthritis: a three-month, multicenter, double-blind, randomized, placebo-controlled study. Arthritis Rheum. 1999;42(7):1386–96.

40. Sohn YB, Cho SY, Park SW, Kim SJ, Ko A-R, Kwon E-K, u. a. Phase I/II clinical trial of enzyme replacement therapy with idursulfase beta in patients with mucopolysaccharidosis II (Hunter syndrome). Orphanet J Rare Dis. 2013;8:42.

41. Specks U, Merkel PA, Seo P, Spiera R, Langford CA, Hoffman GS, u. a. Efficacy of Remission-Induction Regimens for ANCA-Associated Vasculitis. N Engl J Med. 1. August 2013;369(5):417–27.

42. Stone JH, Merkel PA, Spiera R, Seo P, Langford CA, Hoffman GS, u. a. Rituximab versus Cyclophosphamide for ANCA-Associated Vasculitis. N Engl J Med. 15. Juli 2010;363(3):221–32.

43. Stone JH, Tuckwell K, Dimonaco S, Klearman M, Aringer M, Blockmans D, u. a. Trial of Tocilizumab in Giant-Cell Arteritis. N Engl J Med. 27. Juli 2017;377(4):317–28.

44. Tashkin DP, Elashoff R, Clements PJ, Goldin J, Roth MD, Furst DE, u. a. Cyclophosphamide versus placebo in scleroderma lung disease. N Engl J Med. 22. Juni 2006;354(25):2655–66.

45. van de Vlekkert J, Hoogendijk JE, de Haan RJ, Algra A, van der Tweel I, van der Pol WL, u. a. Oral dexamethasone pulse therapy versus daily prednisolone in sub-acute onset myositis, a randomised clinical trial. Neuromuscul Disord. Juni 2010;20(6):382–9.

46. Walter MC, Lochmüller H, Toepfer M, Schlotter B, Reilich P, Schröder M, u. a. High-dose immunoglobulin therapy in sporadic inclusion body myositis: a double-blind, placebo-controlled study. J Neurol. Januar 2000;247(1):22–8.

47. Wechsler ME, Akuthota P, Jayne D, Khoury P, Klion A, Langford CA, u. a. Mepolizumab or Placebo for Eosinophilic Granulomatosis with Polyangiitis. N Engl J Med. 18. Mai 2017;376(20):1921–32.

48. Yurdakul S, Mat C, Tüzün Y, Ozyazgan Y, Hamuryudan V, Uysal O, u. a. A double-blind trial of colchicine in Behçet’s syndrome. Arthritis Rheum. 2001;44(11):2686–92.

49. Distler O, Highland KB, Gahlemann M, Azuma A, Fischer A, Mayes MD, u. a. Nintedanib for Systemic Sclerosis–Associated Interstitial Lung Disease. N Engl J Med. 27. Juni 2019;380(26):2518–28.

50. Hatemi G, Mahr A, Ishigatsubo Y, Song Y-W, Takeno M, Kim D, u. a. Trial of Apremilast for Oral Ulcers in Behçet’s Syndrome. N Engl J Med. 14. November 2019;381(20):1918–28.
